# Neurofibromatose 1: à propos d'un cas historique

**DOI:** 10.11604/pamj.2014.18.191.4904

**Published:** 2014-07-04

**Authors:** Nada El Moussaoui, Nadia Ismaili

**Affiliations:** 1 Service de Dermatologie, CHU Ibn Sina, Université Med V, Souissi, Rabat, Maroc

**Keywords:** Neurofibromatose, maladie de Von Recklinghausen, maladie génétique, neurofibromatosis, Von Recklinghausen disease, genetic disease

## Image en medicine

La neurofibromatose 1 (NF1) ou maladie de Von Recklinghausen est une maladie génétique fréquente. C'est une affection autosomique dominante qui se caractérise par une expression clinique variable au sein d'une même famille. Les sept critères diagnostiques cardinaux sont: l'atteinte d'un apparenté premier degré; au moins six taches café au lait; des lentigines axillaires ou inguinales; au moins deux neurofibromes; un gliome des voies optiques; au moins deux nodules de Lisch et une atteinte osseuse caractéristique. Le diagnostic positif est posé si deux critères sont présents. Les complications de la NF1 sont peu fréquentes mais graves. Elles se présentent sous forme de neurofibromes plexiformes, de gliomes intracérébraux, de tumeurs des gaines nerveuses, d'anomalies vasculaires et de dysplasies osseuses. Un suivi régulier et à vie est nécessaire pour les détecter. Nous rapportons le cas d'un jeune homme de 38 ans, sans antécédent familial particulier, qui présente une NF1 sévère avec une hémiparésie faciale droite. Un scanner cérébro-thoraco-abdomino-pelvien en coupes fines était réalisé qui a objectivé un énorme gliome du tronc cérébral. Le patient est décédé avant tout acte chirurgical.

**Figure 1:**
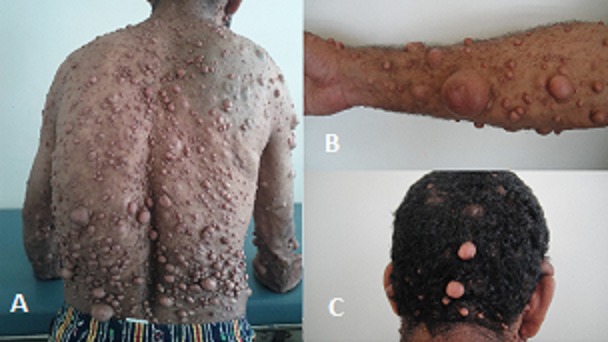
Patient présentant d’énormes neurofibromes au niveau (A) du tronc, (B) avant-bras, et (C) tête

